# Social determinants of health and cardiovascular health across female life stages: Reproductive and midlife women in the National Health Interview Survey

**DOI:** 10.1016/j.ajpc.2026.101552

**Published:** 2026-03-14

**Authors:** Bede N. Nriagu, Shiwani Mahajan, Yaa Adoma Kwapong, Samuel A. Ayedun, Huzaifa Faizan, Muhammad Khalid Tahir, Faith E. Metlock, Lily Dastmalchi, Zulqarnain Javed, Garima Sharma

**Affiliations:** a Inova Schar Heart and Vascular, Inova Health System, Falls Church, VA, USA; b Yale School of Medicine, New Haven, Connecticut, USA; c Johns Hopkins School of Medicine, Baltimore, MD, USA; d Montefiore Medical Center, Bronx, NY, USA; e William Carey College of Osteopathic Medicine, Hattiesburg, Mississippi, USA; f New York Medical College/Metropolitan Hospital Center, New York, NY, USA; g Houston Methodist DeBakey Heart & Vascular Center, Houston Methodist, Houston, TX, USA

**Keywords:** Cardiovascular risk profiles, Social determinants of health, Women’s health, Female life stages

## 1 Introduction

Cardiovascular disease (CVD) remains the leading cause of morbidity and mortality among women in the United States (US), accounting for nearly one in three female deaths [1]. CVD risk emerges early in the life course, with nearly half of women aged 20 years and older living with at least one form of CVD [2]. Despite this burden, conventional cardiovascular risk frameworks incompletely capture broader life-course influences, including the cumulative burden of social determinants of health (SDOH) that shape women's cardiovascular trajectories [3].

Growing evidence demonstrates that adverse SDOH exert durable effects on cardiovascular health (CVH), and women experience a high burden of psychosocial stressors which have been associated with CVD [4]. Although SDOH are increasingly recognized as important drivers of cardiovascular risk, most prior studies have focused on individual determinants rather than cumulative social disadvantage [5]. In this study, we examined the association between cumulative SDOH burden and suboptimal CVH among US women, stratified by reproductive (18–44 years) and midlife (45–60 years) stages.

## 2 Methods

### 2.1 Data source and study population

We analyzed data from the National Health Interview Survey (NHIS), an annual cross-sectional survey conducted by the National Center for Health Statistics that provides nationally representative estimates of the health of the noninstitutionalized US population [6]. NHIS uses a complex multistage sampling design to ensure national representation [6]. Detailed survey structure and variable sources are described in **Supplementary Material**.

**Fig. 1:**
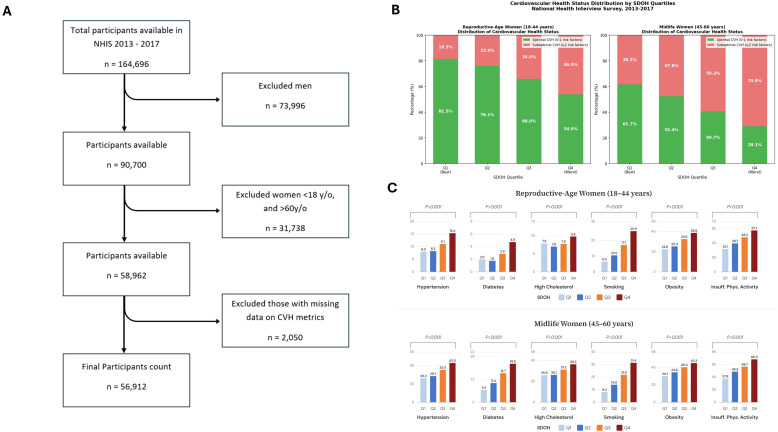
Study population flow diagram National Health Interview Survey (NHIS), 2013–2017 and prevalence of cardiovascular risk factors and optimal versus suboptimal cardiovascular health across SDOH quartiles among reproductive-age and midlife women.

We performed a cross-sectional analysis of pooled NHIS data from 2013–2017. The study population included women aged 18–60 years (Fig. 1). Because menopausal status were not consistently captured in NHIS, age was used as a proxy for life stage, categorizing participants as reproductive-aged (18–44 years) and midlife (45–60 years), consistent with prior literature [5,7].

### 2.2 Exposure and outcome definitions

The primary exposure was cumulative SDOH burden, assessed using a composite score derived from socioeconomic and psychosocial indicators available in NHIS. The score incorporated measures spanning economic stability, education, health care access, food security, neighborhood and social environment, and psychosocial context. Domain definitions are described in **Supplementary Material**.

CVH was defined using six NHIS cardiometabolic risk factors: hypertension, diabetes, hypercholesterolemia, smoking, obesity, and physical inactivity. Diet was excluded due to unavailable data and sleep due to missingness. CVH was categorized as optimal (0–1 risk factors) or suboptimal (≥2) reflecting cumulative cardiometabolic risk factor burden as a proxy for the comprehensive American Heart Association (AHA) CVH construct consistent with prior literature [6]. (**Supplementary Material**).

### 2.3 Statistical analysis

Survey-weighted descriptive statistics were used to generate nationally representative estimates across SDOH quartiles. Multivariable logistic regression models assessed associations between SDOH burden and suboptimal CVH and individual risk factors, adjusting for age and race/ethnicity. Additional socioeconomic variables were excluded in the model to avoid overadjustment, as they were components of the SDOH exposure. All analyses incorporated NHIS sampling weights. Statistical analyses were performed using SAS version 9.4 (SAS Institute Inc).

## 3 Results

The analytic sample included 56,912 women aged 18–60 years (mean age 39.2 ± 11.9 years), representing approximately 86.5 million US women after survey weighting. Of these, 63.8 % were of reproductive age (18–44 years), and 36.2 % were midlife stage (45–60 years). The overall cohort was predominantly non-Hispanic White (59.6 %). Cardiometabolic risk factors, including hypertension, diabetes, hyperlipidemia, obesity, smoking, and insufficient physical activity were significantly more prevalent among midlife women (all *p*<.0001), who also had a higher burden of suboptimal CVH compared to their reproductive-age counterparts [Table 1].

**Table 1:** Baseline characteristics by reproductive age and midlife women - National Health Interview Survey (NHIS), 2013–2017, women aged 18–60 years.

	Overall Characteristics	Reproductive (Age 18–44)	Midlife (45–60)	p-values
N ( %)	56,912 (100)	35,690 (63.8)	21,222 (36.2)	<0.0001
Age (Mean, SD)	39.2 (11.9)	31.5 (7.4)	52.2 (4.3)	<0.0001
SDOH Quartiles				<0.0001
Quartile 1	12,288 (21.6)	7175(20.1)	5113 (24.1)	
Quartile 2	13,251 (23.3)	8567 (24.0)	4684 (22.1)	
Quartile 3	14,420 (25.3)	9333 (26.2)	5087 (24.0)	
Quartile 4	16,953 (29.8)	10,615 (29.7)	6338 (29.9)	
**Race/Ethnicity (N,%)**				<0.0001
Non-Hispanic White	33,420 (59.6)	19,666(55.9)	13,754(65.7)	
Non-Hispanic Black	8670 (15.5)	5463 (15.5)	3207 (15.3)	
Non-Hispanic Asian	3436 (6.1)	2410 (6.9)	1026 (4.9)	
Hispanic	10,593 (18.9)	7648 (21.7)	2945 (14.1)	
				
**CV Risk Factors (N,%)**				
Hypertension	11,178 (19.6)	3958 (11.1)	7220 (34.0)	<0.0001
Diabetes	3459 (6.1)	1068(3.0)	2391 (11.3)	<0.0001
High Cholesterol	9383 (16.5)	2944 (8.3)	6439 (30.3)	<0.0001
Smoking	9903 (17.4)	5725 (16.0)	4178 (19.7)	<0.0001
Obesity	19,041 (33.5)	10,953(30.7)	8088 (38.1)	<0.0001
Insufficient Physical activity	27,757 (48.8)	16,323 (45.7)	11,434 (53.9)	<0.0001
**CVH**				<0.0001
Optimal (0–1)	33,783 (59.4)	24,264 (68.0)	9519 (44.9)	
Suboptimal (>/=2)	23,129 (40.6)	11,426(32.0)	11,703 (55.2)	

When stratified by life stage, higher SDOH burden was associated with progressively greater prevalence of cardiometabolic risk factors in both reproductive-age and midlife women, with midlife women consistently exhibiting a higher overall burden. Correspondingly, suboptimal CVH increased with worsening SDOH burden, from 18.5 % to 38.3 % in reproductive-age women and 46.0 % to 70.9 % in midlife women between the lowest and highest SDOH quartiles (*p*<.0001) **[Supplemental Material].**

In multivariable models adjusted for age and race/ethnicity, greater SDOH disadvantage was independently associated with higher odds of suboptimal CVH and individual cardiovascular risk factors in both life stages. Compared with the most favorable quartile, women in the most disadvantaged quartile had more than threefold higher odds of suboptimal CVH among reproductive-age (OR 3.64, 95 % CI 3.38–3.92) and midlife women (OR 3.34, 95 % CI 3.08–3.63), with graded associations across hypertension, diabetes, obesity, hyperlipidemia, smoking, and physical inactivity **[Supplemental Material]**. No significant interaction was observed between SDOH burden and suboptimal CVH by life stage (*p*=.85), indicating consistent associations across age groups.

### 3.1 Discussion

Our study demonstrates that suboptimal CVH is prevalent among US women across key reproductive life stages, affecting more than half of midlife women and nearly one-third of reproductive-age women. Social disadvantage manifested differently across life stages. Among reproductive-age women, adverse SDOH were most strongly associated with hypertension, obesity, and particularly smoking, suggesting that behavioral and early cardiometabolic pathways may drive the relationship between social disadvantage and cardiovascular risk in early adulthood. In contrast, among midlife women, adverse SDOH were most strongly associated with diabetes, physical inactivity, and hyperlipidemia, with women in the most disadvantaged quartile exhibiting substantially higher odds of these conditions. This likely reflects the interaction between cumulative social disadvantage and metabolic changes during menopause [8], which may accelerate cardiometabolic risk.

Among midlife women, worsening SDOH was associated with worsening prevalence of CVD risk factors and suboptimal CVH. For reproductive-age women, similar patterns were noted except for diabetes and hyperlipidemia, with the second SDOH quartile showing the least prevalence. These patterns may suggest that the relationship between social disadvantage and cardiovascular risk may be modified by life stage. In younger women, moderate social disadvantage may coexist with transient protective factors, such as workforce participation, and stronger social networks, which have been to linked to lower CVD risk [9]. In contrast, extreme social disadvantage may overwhelm these buffers through chronic psychosocial stress or other pathways that have been consistently linked to adverse CVH [10].

These findings have important clinical and public health implications. In reproductive-age women, prevention strategies addressing socially patterned behaviors such as smoking, obesity, and early hypertension may offer substantial long-term benefit, particularly among women facing adverse social conditions. Screening for cumulative social disadvantage and linking women to supportive resources may help reduce early cardiovascular risk trajectories. During the midlife stage, targeted interventions focusing on physical activity, metabolic health, and lipid management may be especially critical as biological vulnerability converges with cumulative social disadvantage.

Our study has several limitations. The cross-sectional design precludes causal inference. Age was used as a proxy for menopausal status and may result in misclassification. NHIS data are self-reported and may be subject to recall bias of both SDOH and cardiovascular risk factors. Analyses were restricted to 2013–2017 due to the major NHIS redesign in 2019 and may not capture contemporary trends in CVH risk factors and SDOH. Additionally, the SDOH score equally weighted components and included factors that may act as mediators (e.g., psychological distress) rather than upstream determinants. CVH measure represents a proxy derived from available NHIS variables and does not include all AHA CVH components. Lastly, statistically significant differences in missing CVH variables across SDOH were noted; exclusion of these observations may introduce selection bias.

## 4 Conclusions

Cumulative social disadvantage is strongly associated with suboptimal CVH among US women across both reproductive and midlife stages. Integrating SDOH-informed screening and interventions into routine care may help improve cardiovascular outcomes across the female life course.

## Sources of funding

None.

## CRediT authorship contribution statement

**Bede N. Nriagu:** Writing – review & editing, Writing – original draft, Visualization, Methodology, Formal analysis, Conceptualization. **Shiwani Mahajan:** Writing – review & editing, Writing – original draft. **Yaa Adoma Kwapong:** Writing – original draft. **Samuel A. Ayedun:** Writing – original draft. **Huzaifa Faizan:** Writing – original draft. **Muhammad Khalid Tahir:** Writing – review & editing. **Faith E. Metlock:** Writing – review & editing. **Lily Dastmalchi:** Writing – review & editing. **Zulqarnain Javed:** Writing – review & editing, Supervision, Conceptualization. **Garima Sharma:** Writing – review & editing, Supervision, Conceptualization.

## Declaration of competing interest

The authors declare that they have no known competing financial interests or personal relationships that could have appeared to influence the work reported in this paper.

## Appendix Supplementary materials

Image, application 1
