# Supplementary material for: Social determinants of health and cardiovascular health across female life stages: Reproductive and midlife women in the National Health Interview Survey
Source: Am J Prev Cardiol. 2026 Mar 14;27:101552. doi: 10.1016/j.ajpc.2026.101552 (PMC13261267; doi:10.1016/j.ajpc.2026.101552)
Supplement: Supplementary file 1 [file mmc1.docx]

**Social Determinants of Health and Cardiovascular Health Across Female Life Stages: Reproductive vs Midlife Women in the National Health Interview Survey**

Bede N. Nriagu^1^, Shiwani Mahajan^2^, Yaa Adoma Kwapong^3^, Samuel A. Ayedun^4^, Huzaifa Faizan^5^, Muhammad Khalid Tahir^6^, Faith E. Metlock^1^, Lily Dastmalchi^1^, Zulqarnain Javed^7^, Garima Sharma^1^

^1^ Inova Schar Heart and Vascular, Inova Health System, Falls Church, VA, USA.

^2^ Yale School of Medicine, New Haven, Connecticut, USA

^3^ Johns Hopkins School of Medicine, Baltimore, MD, USA

^4^ Montefiore Medical Center, Bronx, NY, USA

^5^ William Carey College of Osteopathic Medicine, Hattiesburg, Mississippi, US.

^6^ New York Medical College/Metropolitan Hospital Center, New York, NY, US

^7^ Houston Methodist DeBakey Heart & Vascular Center, Houston Methodist, Houston, TX, US.

**Corresponding Author**:

Garima Sharma MD, FAHA, FACC

Inova Health System

8081 Innovation Drive

Falls Church VA

Garima.Sharma@inova.org

**SUPPLEMENTARY MATERIAL**

**Methods**

We analyzed data from the National Health Interview Survey (NHIS), an annual, cross-sectional survey maintained by the National Center for Health Statistics/Centers for Disease Control and Prevention, which provides key national estimates on the health of individuals living in the U.S. outside institutional settings.[1] The NHIS employs a multistage, complex sampling design, including stratification, clustering, and intentional oversampling, to ensure nationally representative estimates of the U.S. population.[2]

The NHIS survey instrument includes four primary components: Household Composition, Family Core, Sample Child Core, and Sample Adult Core. The Household Composition section documents all members residing within a household, while the Family Core captures family-level sociodemographic and health-related information, including overall health status, functional limitations, injury history, and insurance coverage. Within each participating family, one adult and one child are randomly chosen to complete the Sample Adult and Sample Child questionnaires, which collect more detailed information on health behaviors, chronic conditions, healthcare utilization barriers, and financial challenges. For our analysis, we relied primarily on the Sample Adult Core file and augmented these data with variables from the Household Composition and Family Core files. Because the dataset is publicly accessible and fully de-identified, the study met criteria for exemption from institutional review board oversight. [3]

*Study Design and Population*

We performed a cross-sectional analysis of pooled data from the NHIS between 2013 and 2017. Our study population included women aged 18 to 60 years[Figure 1]. Because menopausal status was not directly measured, age was used as a proxy for life stage, consistent with prior literature, categorizing women as reproductive age (18-44 years)[4] and midlife (45-60 years)[5]

*SDOH Aggregate Score*

We developed a composite SDOH measure incorporating 38 indicators across six domains, based on the Kaiser Family Foundation framework and prior literature on factors influencing CVH.[6] The SDOH score included six domains: economic stability (employment, income, and financial burden related to adherence and cost barriers); neighborhood, physical environment, and social cohesion (house tenure and neighborhood quality); community and social context (psychological distress symptoms: sadness, restlessness/fidgeting, nervousness, hopelessness, worthlessness, and that everything takes effort); education (English proficiency, highest education level, and health literacy measured through use of health information technology); food (food insecurity); and health care (insurance status, usual source of care, delayed/forgone care in accessing health care, and quality of health care).

Binary variables were coded as 0 for favorable and 1 for unfavorable responses. Multi-item or multi-level variables (e.g., the 10-item food insecurity tool) were similarly dichotomized using established cutoffs.[7] Each participant’s SDOH score was calculated as the sum of unfavorable indicators across the six domains (range: 0-38). Quartiles were created as follows: first (score 3-6), second (score 7-9), third (score 10-13), and fourth (score ≥14). The first quartile was defined as the most favorable SDOH profile, whereas the fourth quartile was defined as the most unfavorable SDOH profile.

*Ascertainment of CVH Status*

Cardiovascular risk factors were ascertained through self-reported responses collected during the NHIS household interview and reflect either prior physician diagnoses (e.g., hypertension, diabetes) or current health behaviors/status at the time of the survey. Diet was excluded from the definition similar to prior published literature.[8] Hypertension, diabetes, and hypercholesterolemia were determined from participant self-report, and current smoking status was also based on self-reported behavior. Obesity was defined as a body mass index of ≥30 kg/m². Women were considered to have inadequate physical activity if they did not meet the recommended thresholds of at least 75 minutes of vigorous activity per week, 150 minutes of moderate activity per week, or any equivalent combination totaling ≥150 minutes of moderate-to-vigorous aerobic activity. Because the NHIS does not capture dietary intake, diet could not be incorporated into the CVH score. In addition, sleep was excluded from the CVH definition because 79% of the study population had missing sleep duration data. Cardiovascular health (CVH) was operationalized as a proxy measure based on available NHIS variables, reflecting the burden of traditional cardiometabolic risk factors. Accordingly, CVH was categorized as optimal if there are 0-1 risk factors and suboptimal if ≥2 risk factors based on previously published literature [8, 9].

*Statistical Analysis*

We applied survey-weighted descriptive statistics to generate nationally representative estimates across SDOH quartiles. Categorical data were summarized as weighted counts and percentages, while continuous variables were reported as weighted means and standard deviations. Age-adjusted prevalence estimates for suboptimal CVH and cardiovascular risk factors were calculated overall and by SDOH quartiles, using direct standardization to the 2000 U.S. Census population.[10] To examine the association between SDOH burden and cardiovascular outcomes, multivariable logistic regression models were used to estimate associations with individual risk factors and overall cardiovascular health, adjusting for age and race/ethnicity as demographic confounders. Additional socioeconomic variables were not included to avoid overadjustment because these factors were components of the SDOH exposure.

SDOH exposure was modeled in quartiles across risk factors and suboptimal CVH. All analyses incorporated survey design features, including person-level weights and variance estimation methods, consistent with NHIS’s complex sampling structure. Variance estimates for the pooled dataset were based on the Integrated Public Use Microdata Series. [11] Statistical analyses were conducted using SAS 9.4 (SAS Institute Inc., Cary, NC).

**Table S1: Definition of Social Determinants of Health (SDOH), by Domain**

| Supplemental Table 1. Social determinants of health components used, by domain. | | | |
| --- | --- | --- | --- |
| ***Short version of items*** | ***Long version of items*** | ***Survey Responses*** | ***Analytic recode*** |
| **ECONOMIC STABILITY** | | | |
| Employment | What was your employment status as of last week? | Working for pay at a job or business; With a job or business but not at work; Looking for work; Working, but not for pay, at a family-owned job or business; Not working at a job or business and not looking for work | 0 = "Employed or Retired"; 1 = "Never or Previously Employed" |
| Sick Leave | Paid sick leave at current job or most current job | Yes; No | 0 = "Yes"; 1 = "No" |
| Family Income | Ratio of family income to poverty threshold |  | 0 = "Middle/High-income" (≥ 200% of poverty threshold); 1 = "Low-income" (< 200% of poverty threshold) |
| Difficulty Paying Medical Bills | In the past 12 months did you/anyone in the family have problems paying or were unable to pay any medical bills? Include bills for doctors, dentists, hospitals, therapists, medication, equipment, nursing home or home care. | Yes; No | 0 = "No"; 1 = "Yes" |
| Unable to Pay Medical Bills | If previous question = Yes: Do you/Does anyone in your family currently have any medical bills that you are unable to pay at all? | Yes; No | 0 = "No"; 1 = "Yes" |
| High Financial Distress Composite Score (aggregate score from the following 6 questions);  Worried about … | | | From the aggregate sum of the following 6 items, divided into quartiles:  0 = quartiles 1-3; 1 = quartile 4 |
| … Money for retirement | How worried are you right now about not having enough money for retirement? | Very worried; Moderately worried; Not too worried; Not worried at all | 0 = "Not too worried/Not worried at all"; 1 = "Mod/Very worried" |
| … Medical costs of illness/accident | How worried are you right now about not being able to pay medical costs of a serious illness or accident? | Very worried; Moderately worried; Not too worried; Not worried at all | 0 = "Not too worried/Not worried at all"; 1 = "Mod/Very worried" |
| … Maintaining standard of living | How worried are you right now about not being able to maintain the standard of living you enjoy? | Very worried; Moderately worried; Not too worried; Not worried at all | 0 = "Not too worried/Not worried at all"; 1 = "Mod/Very worried" |
| … Medical costs of healthcare | How worried are you right now about not being able to pay medical costs for normal healthcare? | Very worried; Moderately worried; Not too worried; Not worried at all | 0 = "Not too worried/Not worried at all"; 1 = "Mod/Very worried" |
| … Paying monthly bills | How worried are you right now about not having enough to pay your normal monthly bills? | Very worried; Moderately worried; Not too worried; Not worried at all | 0 = "Not too worried/Not worried at all"; 1 = "Mod/Very worried" |
| … Paying rent/mortgage/housing costs | How worried are you right now about not being able to pay your rent, mortgage, or other housing costs? | Very worried; Moderately worried; Not too worried; Not worried at all | 0 = "Not too worried/Not worried at all"; 1 = "Mod/Very worried" |
| Cost-related medication non-adherence (positive if any of the following 3 questions' answer is Yes): |  |  |  |
| … Skipped medication doses to save money | DURING THE PAST 12 MONTHS, were any of the following true for you? …You skipped medication doses to save money | Yes; No | 0 = "No"; 1 = "Yes" |
| … Took less medicine to save money | DURING THE PAST 12 MONTHS, were any of the following true for you? …you took less medicine to save money | Yes; No | 0 = "No"; 1 = "Yes" |
| … Delayed filling prescription to save money | DURING THE PAST 12 MONTHS, were any of the following true for you? …You delayed filling a prescription to save money | Yes; No | 0 = "No"; 1 = "Yes" |
| Delayed Care due to Cost | DURING THE PAST 12 MONTHS, has medical care been delayed because of worry about the cost? (Do not include dental care) | Yes; No | 0 = "No"; 1 = "Yes" |
| Foregone Care due to Cost | DURING THE PAST 12 MONTHS, was there any time when (31) needed medical care, but did not get it because (31) couldn't afford it? | Yes; No | 0 = "No"; 1 = "Yes" |
| **NEIGHBOURHOOD, PHYSICAL ENVIRONMENT AND SOCIAL COHESION** | | | |
| House Tenure | Is this house/apartment owned or being bought, rented, or occupied by some other arrangement by [you/or someone in your family]? | Owned or being bought; Rented; Other arrangement | 0 = "Own or being bought"; 1 = "Rent/Other arrangement" |
| Neighborhood Quality (Help) | How much do you agree or disagree with the following statements about your neighborhood? Would you say… People in this neighborhood help each other out. | Definitely agree; Somewhat agree; Somewhat disagree; Definitely disagree | 0 = "Agree (Somewhat/Definitely)"; 1 = "Disagree (Somewhat/Definitely)" |
| Neighborhood Quality (Trust) | How much do you agree or disagree with the following statements about your neighborhood? Would you say… People in this neighborhood can be trusted. | Definitely agree; Somewhat agree; Somewhat disagree; Definitely disagree | 0 = "Agree (Somewhat/Definitely)"; 1 = "Disagree (Somewhat/Definitely)" |
| Neighborhood Quality (Close Knit) | How much do you agree or disagree with the following statements about your neighborhood? Would you say… This is a close-knit neighborhood. | Definitely agree; Somewhat agree; Somewhat disagree; Definitely disagree | 0 = "Agree (Somewhat/Definitely)"; 1 = "Disagree (Somewhat/Definitely)" |
| Neighborhood Quality (Accountability) | How much do you agree or disagree with the following statements about your neighborhood? Would you say… There are people I can count on in this neighborhood. | Definitely agree; Somewhat agree; Somewhat disagree; Definitely disagree | 0 = "Agree (Somewhat/Definitely)"; 1 = "Disagree (Somewhat/Definitely)" |
| **COMMUNITY AND SOCIAL CONTEXT** | | | |
| Kessler K6 Scale for High Psychological Distress (derived from the following 6 questions): | | | From the aggregate sum of the following 6 items:  0 = "No psychological distress" (sum ≤ 12); 1 = "Psychological distress" (sum ≥ 13) |
| … Feeling sad | During the past 30 days, how often did you feel …so sad that nothing could cheer you up? | All of the time; Most of the time; Some of the time; A little of the time; None of the time | 0 = "None of the time"; 1 = "A little of the time"; 2 = "Some of the time"; 3 = "Most of the time"; 4 = "All of the time" |
| … Nervous | During the past 30 days, how often did you feel … nervous? | All of the time; Most of the time; Some of the time; A little of the time; None of the time | 0 = "None of the time"; 1 = "A little of the time"; 2 = "Some of the time"; 3 = "Most of the time"; 4 = "All of the time" |
| … Restless/fidgety | During the past 30 days, how often did you feel … restless or fidgety? | All of the time; Most of the time; Some of the time; A little of the time; None of the time | 0 = "None of the time"; 1 = "A little of the time"; 2 = "Some of the time"; 3 = "Most of the time"; 4 = "All of the time" |
| … Hopeless | During the past 30 days, how often did you feel … hopeless? | All of the time; Most of the time; Some of the time; A little of the time; None of the time | 0 = "None of the time"; 1 = "A little of the time"; 2 = "Some of the time"; 3 = "Most of the time"; 4 = "All of the time" |
| … Everything was an effort | During the past 30 days, how often did you feel … that everything was an effort? | All of the time; Most of the time; Some of the time; A little of the time; None of the time | 0 = "None of the time"; 1 = "A little of the time"; 2 = "Some of the time"; 3 = "Most of the time"; 4 = "All of the time" |
| … Worthless | During the past 30 days, how often did you feel … worthless? | All of the time; Most of the time; Some of the time; A little of the time; None of the time | 0 = "None of the time"; 1 = "A little of the time"; 2 = "Some of the time"; 3 = "Most of the time"; 4 = "All of the time" |
| **FOOD** | | | |
| Food Insecurity (based on US Dept. of Agriculture Standardized Questionnaire) | | | From the aggregate sum of the following 10 items:  0 = "Food Secure" (sum ≤ 2); 1 = "Food Insecure" (sum ≥ 3) |
| … Worried food would run out before got money to buy more | [fill 2: I/We] worried whether [fill 3: my/our] food would run out before [fill 4: I/we] got money to buy more. Was that often true, sometimes true, or never true for [fill 1: you/your family] in the last 30 days? | Often true; Sometimes true; Never true | 0 = "Never true"; 1 = "Sometimes true/Often true" |
| … Food did not last before had money to get more | The food that [fill 1: I/we] bought just didn't last, and [fill 1: I/we] didn't have money to get more. Was that often true, sometimes true, or never true for [fill 2: you/your family] in the last 30 days? | Often true; Sometimes true; Never true | 0 = "Never true"; 1 = "Sometimes true/Often true" |
| … Could not afford to eat balanced meals | [fill 1: I/We] couldn't afford to eat balanced meals. Was that often true, sometimes true, or never true for [fill 2: you/your family] in the last 30 days? | Often true; Sometimes true; Never true | 0 = "Never true"; 1 = "Sometimes true/Often true" |
| … Cut size or skipped meals because not enough money | In the last 30 days, did [fill 1: you/you or other adults in your family] ever cut the size of your meals or skip meals because there wasn't enough money for food? | Yes; No | 0 = "No"; 1 = "Yes" |
| … If above question = Yes: How many days in past month? | In the last 30 days, how many days did this happen? | 01-30 days (continuous response) | 0 = if < 3 days; 1 = if ≥ 3 days |
| … Eat less than felt should because not enough money | In the last 30 days, did you ever eat less than you felt you should because there wasn't enough money for food? | Yes; No | 0 = "No"; 1 = "Yes" |
| … Hungry but did not eat because not enough money | In the last 30 days, were you ever hungry but didn't eat because there wasn't enough money for food? | Yes; No | 0 = "No"; 1 = "Yes" |
| … Lose weight because not enough money for food | In the last 30 days, did you lose weight because there wasn't enough money for food? | Yes; No | 0 = "No"; 1 = "Yes" |
| … Not eat for a whole day because not enough money for food | In the last 30 days, did [fill 1: you/you or other adults in your family] ever not eat for a whole day because there wasn't enough money for food? | Yes; No | 0 = "No"; 1 = "Yes" |
| … If above question = Yes: How many days in past month? | In the last 30 days, how many days did this happen? | 01-30 days (continuous response) | 0 = if < 3 days; 1 = if ≥ 3 days |
| **EDUCATION** | | | |
| English Language | How well do you speak English? | Very well; Well; Not well; Not at all | 0 = "Well/Very Well"; 1 = "Not well/Not at all" |
| Education Attainment | What is the HIGHEST level of school completed or the highest degree received? | Never attended/kindergarten only; 1st grade; 2nd grade; 3rd grade; 4th grade; 5th grade; 6th grade; 7th grade; 8th grade; 9th grade; 10th grade; 11th grade; 12th grade; GED or equivalent; High school graduate; Some college, no degree; Associate degree: occupational, technical, or vocational program; Associate degree: academic program; Bachelor's degree; Master's degree; Professional school degree; Doctoral degree | 0 = "≥ Some college"; 1 = "≤ High School" |
| Health Information Technology use: Looked up health info on internet | DURING THE PAST 12 MONTHS, have you ever used computers for any of the following …Look up health information on the Internet | Yes; No | 0 = "No"; 1 = "Yes" |
| Health Information Technology use: Filled a prescription online | DURING THE PAST 12 MONTHS, have you ever used computers for any of the following …Fill a prescription | Yes; No | 0 = "No"; 1 = "Yes" |
| Health Information Technology use: Scheduled a healthcare appointment online | DURING THE PAST 12 MONTHS, have you ever used computers for any of the following …Schedule an appointment with a health care provider | Yes; No | 0 = "No"; 1 = "Yes" |
| Health Information Technology use: Communicated with healthcare provider online | DURING THE PAST 12 MONTHS, have you ever used computers for any of the following …Communicate with a health care provider by email | Yes; No | 0 = "No"; 1 = "Yes" |
| Health Information Technology use: Used internet chat rooms to learn about health topics | DURING THE PAST 12 MONTHS, have you ever used computers for any of the following …Use online chat groups to learn about health topics | Yes; No | 0 = "No"; 1 = "Yes" |
| **HEALTHCARE SYSTEM** | | | |
| Insurance Status | Multiple questions | Uninsured; Private; Medicaid; Medicare; Other | 0 = "Uninsured"; 1 = "Insured" |
| Usual Source of Care | Is there a place that you USUALLY go to when you are sick or need advice about your health? | Yes; There is no place; There is more than one place | 0 = "Usual source of care"; 1 = "No usual source of care" |
| Trouble finding a doctor/provider, past 12m | DURING THE PAST 12 MONTHS, did you have any trouble finding a general doctor or provider who would see you? | Yes; No | 0 = "No"; 1 = "Yes" |
| MD's office not accept you as new patient, past 12m | DURING THE PAST 12 MONTHS, were you told by a doctor’s office or clinic that they would not accept you as a new patient? | Yes; No | 0 = "No"; 1 = "Yes" |
| MD's office not accept your insurance, past 12m | DURING THE PAST 12 MONTHS, were you told by a doctor’s office or clinic that they did not accept your health care coverage? | Yes; No | 0 = "No"; 1 = "Yes" |
| Delayed Medical Care: Couldn't get through on phone | There are many reasons people delay getting medical care. Have you delayed getting care for any of the following reasons in the PAST 12 MONTHS? ..... You couldn't get through on the telephone | Yes; No | 0 = "No"; 1 = "Yes" |
| Delayed Medical Care: Couldn't get appt soon enough | There are many reasons people delay getting medical care. Have you delayed getting care for any of the following reasons in the PAST 12 MONTHS? ..... You couldn't get an appointment soon enough | Yes; No | 0 = "No"; 1 = "Yes" |
| Delayed Medical Care: Wait too long at MD's office | There are many reasons people delay getting medical care. Have you delayed getting care for any of the following reasons in the PAST 12 MONTHS? ..... Once you get there, you have to wait too long to see the doctor | Yes; No | 0 = "No"; 1 = "Yes" |
| Delayed Medical Care: Not open when you could go | There are many reasons people delay getting medical care. Have you delayed getting care for any of the following reasons in the PAST 12 MONTHS? ..... The clinic/doctor's office wasn't open when you could get there | Yes; No | 0 = "No"; 1 = "Yes" |
| Delayed Medical Care: No transportation | There are many reasons people delay getting medical care. Have you delayed getting care for any of the following reasons in the PAST 12 MONTHS? ..... You didn't have transportation | Yes; No | 0 = "No"; 1 = "Yes" |
| Quality of Care (Satisfaction) | In general, how satisfied are you with the healthcare you received in the past 12 months? | Very satisfied; Somewhat satisfied; Somewhat dissatisfied; Very dissatisfied; You haven't had health care in the past 12 months | 0 = "Somewhat/Very Satisfied"; 1 = "Somewhat/Very Dissatisfied or No healthcare in past year" |

**Table S2: Characteristics of the study population by social determinants of health, United States, National Health Interview Survey, 2013-2017, women aged 18-60 years**

|  |  | Social Determinant of Health (SDOH) Quartiles | | | |  |
| --- | --- | --- | --- | --- | --- | --- |
|  | **Overall** | **Quartile 1** | **Quartile 2** | **Quartile 3** | **Quartile 4** | p-value |
| **Sample (N, %)** | 56912  (100) | 12288  (23.8) | 13251  (24.1) | 14420  (25.5) | 16953  (26.6) | <.0001 |
| **Weighted Sample (N, %)** | 86459885 | 20603294 | 20829396 | 22037143 | 22990052 |  |
| **Mean Age (SD), years** | 39.2 (11.9) | 41.1 (11.3) | 38.2 (12.3) | 38.3 (12.1) | 39.4 (11.5) |  |
| **Age Category (Mean, SD), years** |  |  |  |  |  | p<.0001 |
| 18-44 | 31.5 (7.4) | 33.0 (6.9) | 30.5 (7.7) | 30.8 (7.5) | 31.8 (7.1) |  |
| 45-60 | 52.2 (4.3) | 52.4 (4.3) | 52.2 (4.4) | 52.2 (4.3) | 52.0 (4.3) |  |
| **Race/Ethnicity (N, %)** |  |  |  |  |  | p<.0001 |
| Non-Hispanic White | 33420  (59.6) | 9152  (75.2) | 8870  (67.8) | 8047  (56.7) | 7351  (44.1) |  |
| Non-Hispanic Black | 8670  15.5 | 1147  (9.4) | 1599  (12.2) | 2484  (17.5) | 3440  (20.6) |  |
| Non-Hispanic Asian | 3436  (6.1) | 901  (7.4) | 994  (7.6) | 919  (6.5) | 622  (3.7) |  |
| Hispanic | 10593  (18.9) | 973  (8.0) | 1624  (12.4) | 2740  (19.3) | 5256  (31.5) |  |
| **CV Risk Factors (N, %)** |  |  |  |  |  |  |
| Hypertension | 11178  19.6 | 1922  (15.6) | 2053  (15.5) | 2841  (19.7) | 4362  (25.7) | p<.0001 |
| Diabetes | 3459  (6.1) | 421  (3.4) | 546  (4.1) | 918  (6.4) | 1574  (9.3) | p<.0001 |
| High Cholesterol | 9383  (16.5) | 1896  (15.4) | 1828  (13.8) | 2316  (16.1) | 3343  (19.7) | p<.0001 |
| Smoking | 9903  (17.4) | 891  (7.3) | 1558  (11.8) | 2710  (18.8) | 4744  (28.0) | p<.0001 |
| Obesity | 19041  (33.5) | 3162  (25.7) | 3800  (28.7) | 5098  (35.4) | 6981  (41.2) | p<.0001 |
| Insufficient Phys. activity | 27757  (48.8) | 4233  (34.5) | 5683  (42.9) | 7388  (51.2) | 10453  (61.7) | p<.0001 |
| **CVH** |  |  |  |  |  | p<.0001 |
| Optimal (0-1) | 33783  (59.4) | 9001  (73.3) | 8971  (67.7) | 8233  (57.1) | 7578  (44.7) |  |
| Suboptimal (>/=2) | 23129  (40.6) | 3287  (26.8) | 4280  (32.3) | 6187  (42.9) | 9375  (55.3) |  |

**Table S3. Cardiovascular Risk Factors and Health by social determinants of health, across reproductive-age (18-44) and Midlife (45-60) women. United States, National Health Interview Survey, 2013–2017**

|  | Reproductive-age women (18-44 years old) | | | | | p-value | Midlife age women (45-60 years old) | | | | | p-value |
| --- | --- | --- | --- | --- | --- | --- | --- | --- | --- | --- | --- | --- |
|  |  | Social Determinant of Health (SDOH) Quartiles(Q) | | | |  |  | Social Determinant of Health (SDOH) Quartiles(Q) | | | |  |
|  | **Overall** | **Q1** | **Q2** | **Q3** | **Q4** |  | **Overall** | **Q1** | **Q2** | **Q3** | **Q4** |  |
| **CV Risk Factors (N, %)** |  |  |  |  |  |  |  |  |  |  |  |  |
| Hypertension | 3958  11.1 | 577  8.04 | 710  8.29 | 1036  11.10 | 1635  15.40 | <.0001 | 7220  34.02 | 1345  26.31 | 1343  28.67 | 1805  35.48 | 2727  43.03 | <.0001 |
| Diabetes | 1068  2.99 | 142  1.98 | 152  1.77 | 270  2.89 | 504  4.75 | <.0001 | 2391  11.27 | 279  5.46 | 394  8.41 | 648  12.74 | 1070  16.88 | <.0001 |
| High Cholesterol | 2944  8.25 | 569  7.93 | 603  7.04 | 729  7.81 | 1043  9.83 | <.0001 | 6439  30.34 | 1327  25.95 | 1225  26.15 | 1587  31.20 | 2300  36.29 | <.0001 |
| Smoking | 5725  16.04 | 470  6.55 | 903  10.54 | 1598  17.12 | 2754  25.94 | <.0001 | 4178  19.69 | 421  8.23 | 655  13.98 | 1112  21.86 | 1990  31.40 | <.0001 |
| Obesity | 10953  30.69 | 1622  22.61 | 2177  25.41 | 3041  32.58 | 4113  38.75 | <.0001 | 8088  38.11 | 1540  30.12 | 1623  34.65 | 2057  40.44 | 2868  45.25 | <.0001 |
| Insufficient Phys. activity | 16323  45.74 | 2300  32.06 | 3398  39.66 | 4502  48.24 | 6123  57.68 | <.0001 | 11434  53.88 | 1933  37.81 | 2285  48.78 | 2886  56.73 | 4330  68.32 | <.0001 |
| **CVH** |  |  |  |  |  | <.0001 |  |  |  |  |  | <.0001 |
| Optimal (0-1) | 24264  67.99 | 5847  81.49 | 6519  76.09 | 6163  66.03 | 5735  54.03 |  | 9519  44.85 | 3154  61.69 | 2452  52.35 | 2070  40.69 | 1843  29.08 |  |
| Suboptimal (>/=2) | 11426  32.01 | 1328  18.51 | 2048  23.91 | 3170  33.97 | 4880  45.97 |  | 11703  55.15 | 1959  38.31 | 2232  47.65 | 3017  59.31 | 4495  70.92 |  |

**Figure S1: Adjusted odds ratios for individual cardiovascular risk factors and suboptimal cardiovascular health across SDOH quartiles, stratified by reproductive and midlife stages.**


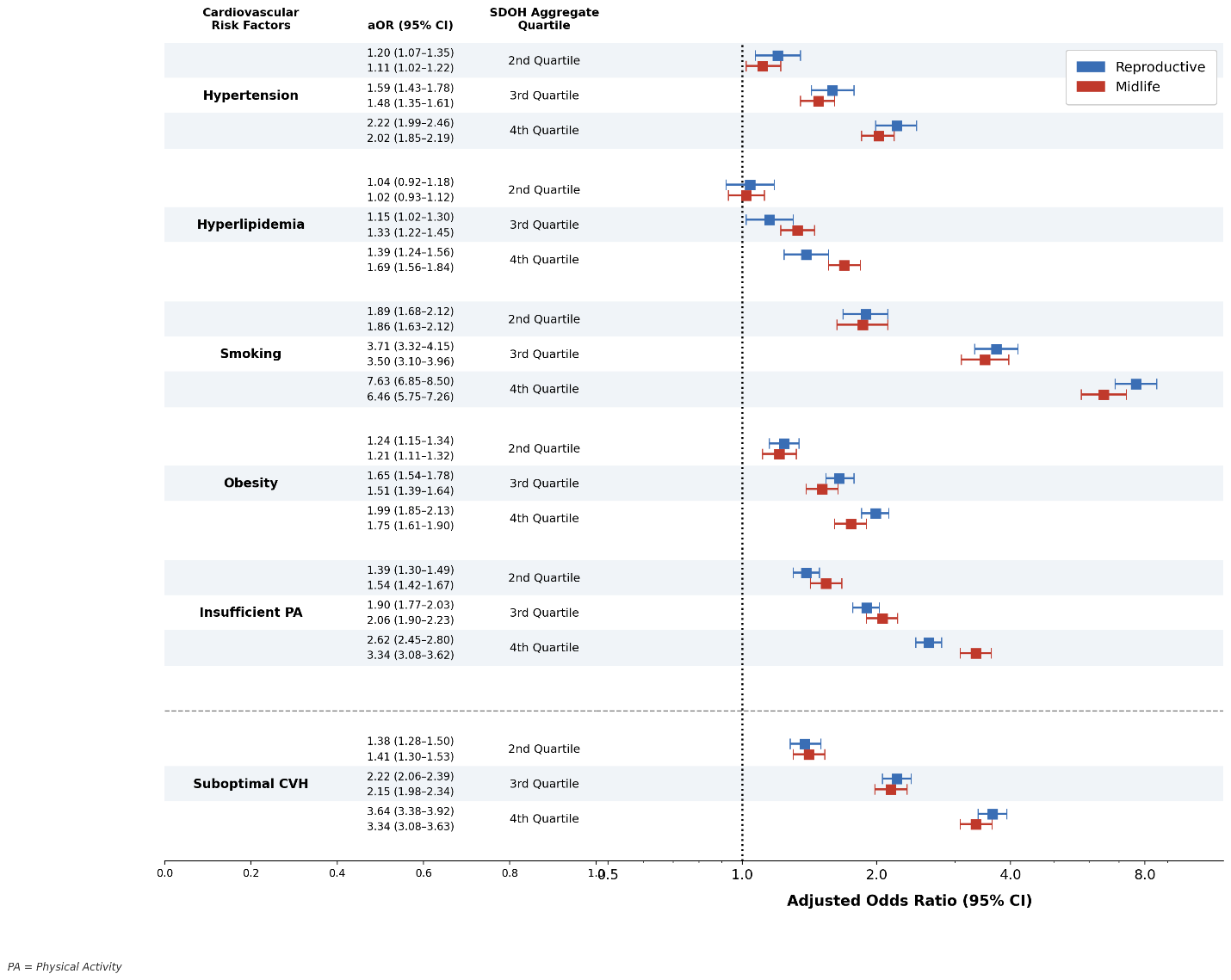


Reference group: SDOH Quartile 1. Odds ratio adjusted for age and race/ethnic group

References

1. National Center for Health Statistics. Available at: https://www.cdc.gov/nchs/nhis/about_nhis.htm. Accessed December 6th, 2025.
2. NHIS data, questionnaires and related documentation. Available at: https://www.cdc.gov/nchs/nhis/documentation/index.html. Accessed December 6th, 2025.
3. IRB Exemption. Available from: https://www.hhs.gov/ohrp/regulations-and-policy/decision-charts-2018/index.html http://www.hhs.gov/ohrp/regulations-andpolicy/decision-trees/. Accessed December 24th, 2025.
4. Boakye, E., et al., Cardiovascular Risk Profile Among Reproductive-Aged Women in the U.S.: The Behavioral Risk Factor Surveillance System, 2015-2020. AJPM Focus, 2024. **3**(4): p. 100210.
5. Harlow, S.D., et al., Women's midlife health: the unfinished research agenda. Womens Midlife Health, 2023. **9**(1): p. 7.
6. Artiga S, Hinton E. Beyond health care: the role of social determinants in promoting health and health equity. Available at: https://www.kff.org/racial-equity-and-health-policy/issue-brief/beyond-healthcare-the-role-of-social-determinants-in-promoting-health-and-healthequity/. Accessed December 27th, 2025.
7. USDA Economic Research Service. Available at: https://www.ers.usda.gov/topics/food-nutrition-assistance/food-security-in-the-us/measurement/#survey. Accessed December 27th, 2026
8. Sharma, G., et al., Social Determinants of Suboptimal Cardiovascular Health Among Pregnant Women in the United States. J Am Heart Assoc, 2022. 11(2): p. e022837.
9. Metlock, F.E., et al., Association between polysocial risk score and CVH among women of reproductive age in the SAFE HEART study: An American Heart Association Research Goes Red Initiative. Curr Probl Cardiol, 2025. 50(3): p. 102947.
10. Age standardization and population estimates. Available at: https://Available from: https://wwwn.cdc.gov/nchs/nhane s/tutor ials/modul e8.aspx. Accessed December 27, 2025
11. Blewett LA, R.D.J., Griffin R, King ML, Williams KCW. IPUMS Health Surveys: National Health Interview Survey, version 6.2 [dataset]. 2017; Available from: doi:10.18128/D070.V6.2.
